# Retrieval Analysis of Sequentially Annealed Highly Crosslinked Polyethylene Used in Total Hip Arthroplasty

**DOI:** 10.1007/s11999-014-4113-9

**Published:** 2014-12-25

**Authors:** Steven M. Kurtz, Daniel W. MacDonald, Michael A. Mont, Javad Parvizi, Arthur L. Malkani, William Hozack

**Affiliations:** 1 Implant Research Center, Drexel University, 3401 Market Street, Suite 345, Philadelphia, PA 19104 USA; 2 Exponent, Inc., 3440 Market Street, Suite 600, Philadelphia, PA 19104 USA; 3 Sinai Hospital of Baltimore, Baltimore, MD USA; 4 Jewish Hospital and St Mary’s Health Care, Louisville, KY USA; 5 Rothman Institute, Philadelphia, PA USA

## Abstract

**Background:**

First-generation annealed and second-generation sequentially annealed, highly crosslinked polyethylenes (HXLPEs) have documented reduced clinical wear rates in their first decade of clinical use compared with conventional gamma inert-sterilized polyethylene. However, for both types of annealed HXLPE formulations, little is known about their reasons for revision, their in vivo oxidative stability, and their resistance to mechanical degradation.

**Questions/purposes:**

We asked whether retrieved sequentially annealed HLXPE acetabular liners exhibited: (1) similar reasons for revision; (2) lower oxidation; (3) improved resistance to wear and degradation of mechanical properties; and (4) improved resistance to macroscopic evidence of rim damage when compared with acetabular liners fabricated from single-dose annealed HXLPE.

**Methods:**

One hundred eighty-five revised acetabular liners in two cohorts (annealed and sequentially annealed) were collected in a multicenter retrieval program between 2000 and 2013. We controlled for implantation time between the two cohorts by excluding annealed liners with a greater implantation time than the longest term sequentially annealed retrieval (5 years); the mean implantation time (± SD) for the annealed components was 2.2 ± 1.4 years, and for the sequentially annealed liners, it was 1.2 ± 1.2 years. Reasons for revision were assessed based on medical records, radiographs, and examinations of the retrieved components. Oxidation was measured at the bearing surface, the backside surface, the locking mechanism, and the rim using Fourier transform infrared spectroscopy (ASTM F2102). Penetration was measured directly using a micrometer (accuracy: 0.001 mm). Mechanical behavior (ultimate load) was measured at the superior and inferior bearing surfaces using the small punch test (ASTM F2183). We used nonparametric statistical testing to analyze for differences in oxidation, penetration rates, and ultimate load when adjusting for HXLPE formulation as a function of implantation time.

**Results:**

The acetabular liners in both cohorts were revised most frequently for instability, loosening, and infection. Oxidation indices (OIs) of the sequentially annealed liners were lower than annealed liners at the bearing surface (mean OI difference = 0.3; p < 0.001), the backside surface (mean OI difference = 0.2; p < 0.001), and the rim (mean OI difference = 2.6; p < 0.001). No differences were detected in linear penetration rates between the cohorts (p = 0.10). Ultimate strength at the bearing surface of the HLXPE was not different between sequentially annealed and annealed cohorts (p = 0.72).

**Conclusions:**

We observed evidence of in vivo oxidation in retrieved annealed and, to a lesser extent, retrieved sequentially annealed acetabular liners. However, we observed no association between the levels of oxidation and clinical performance of the liners.

**Clinical Relevance:**

The findings of this study document the oxidative and mechanical behavior of sequentially annealed HXLPE. The reduced oxidation levels in sequentially annealed liners support the hypothesis that annealing in sequential steps eliminates more free radicals. However, as a result of the short-term followup, analysis of longer-term retrievals is warranted.

## Introduction

In the late 1990s, highly crosslinked polyethylenes (HXLPEs) were clinically introduced for THAs to improve wear resistance of the polyethylene liners and, thus, to reduce the incidence of polyethylene wear debris-induced osteolysis [14]. A secondary goal was to reduce the oxidation of the polymer using thermal treatments. One approach, known as annealing, heats the material to just under the melting point, which reduces the residual free radicals that may promote oxidation; this approach was used with Crossfire (Stryker Orthopedics, Mahwah, NJ, USA), which was clinically introduced in 1998 [17]. Although the residual free radicals have been reduced with this method, they have not been completely eliminated [17]. Over the past 10 years, several studies have reported that annealed HXLPE liners have resulted in reduced wear in THA [7, 20, 21]. However, oxidative degradation occurred with this material, particularly at the exposed polyethylene rim surface [5, 13, 23]. One proposed solution to the in vivo oxidation observed with annealed HXLPE is to perform the annealing in smaller, sequential steps. Aptly known as sequential annealing, the irradiation and annealing process is repeated three times [8, 22]. Clinically introduced in 2005, one sequentially irradiated and annealed HXLPE formulation (X3; Stryker Orthopedics) has been reported to allow for more free radical mobility and thus to more effectively quench free radicals when compared with single-step annealing [8, 22].

Recently, there has been one report of oxidation of sequentially annealed HLXPE. Currier et al. [6] investigated the in vivo oxidation of two remelted (ie, thermal treating above the melting point to eliminate free radicals) HXLPE formulations (Prolong^®^, Zimmer, Warsaw, IN, USA; and XLK, DePuy, Warsaw, IN, USA) and one sequentially annealed HXLPE in TKA. Currier et al. found that both remelted and sequentially annealed HXLPE oxidized in vivo. This was particularly true at the bearing surface where they found that both sequentially annealed and remelted HXLPE exhibited a correlation of oxidation with implantation time. However, the sequentially annealed inserts had higher oxidation rates when compared with remelted HXLPEs. It remains unclear how the oxidation levels in sequentially annealed HLXPE compare with annealed HXLPE in THAs.

The purpose of this multicenter study was to assess the reasons for revision, oxidation, wear, mechanical behavior, and rim damage of second-generation sequentially annealed HXLPE retrieved acetabular liners and to compare these with first-generation annealed HXLPE retrieved acetabular liners as a control. Specifically, we asked whether retrieved sequentially annealed HLXPE acetabular liners exhibited: (1) similar reasons for revision; (2) lower oxidation; (3) improved resistance to wear and degradation of mechanical properties; and (4) improved resistance to macroscopic evidence of rim damage when compared with acetabular liners fabricated from annealed HXLPE.

## Materials and Methods

### Study Design, Cohort Selection, and Clinical Information

Acetabular liners were retrieved during revision surgeries at 10 surgical centers and continuously analyzed between 2000 and 2013 in a prospective, multicenter study of THA component material properties and outcomes. The respective institutional review boards approved the study protocols at all participating centers. Explanted liners were cleaned using institutional procedures and expeditiously stored in a subzero freezer to minimize ex vivo oxidative changes, as described previously [13, 16].

During the study period, our retrieval program received approximately 2700 liners, of which 122 liners were confirmed as being fabricated from a single sequentially annealed HXLPE (X3; Stryker Orthopaedics) and a single design (Trident; Stryker Orthopaedics). Four additional sequentially annealed liners that had been retrieved and were used in conjunction with a mobile bearing design were excluded from the study. Thus, all Trident X3 components received at our institution during the study period were analyzed in this study. X3 is fabricated from compression-molded GUR 1020 polyethylene stock material, which has been gamma-irradiated and annealed in three steps, each with 30 kGy, for a total absorbed dose of 90 kGy [22]. The irradiated stock material is machined into liners and terminally sterilized using gas plasma [22]. The sequentially annealed liners were implanted for a mean ± SD of 1.2 ± 1.2 years (range, 0–5 years). Sterilization information was traceable by the manufacturer from the lot codes in 106 of 122 (88%) retrieved liners. The mean shelf life of the retrievals was 0.6 ± 1.0 years (range, 0.0–4.7 years).

Retrieved liners from the study cohort had inner diameters ranging from 22 to 44 mm (median, 36 mm). The head material was a cobalt-chrome alloy in 66% (80 of 122) of the cases and a ceramic in 33% (40 of 122) of the cases (Biolox^®^ Delta [Ceramtec, Plochingen, Germany] in 35, alumina in four, and oxinium in one). There were 2% (two) of the cases in which the femoral head was not revised and the material was not ascertainable from the revision operative notes. The measured thickness of the liners, in unworn locations, ranged from 4 to 13 mm. The outer diameter of the acetabular shells varied between 44 and 70 mm (median, 54 mm).

Because one of the main goals of our study was to examine in vivo oxidation, which may vary with implantation time, we matched our control cohort to the study cohort by including only those control liners with an implantation time that did not exceed the maximum implantation time (5 years) in the study cohort. The control cohort represented an update of a previous study investigating 60 annealed HXLPE liners, 49 of which were implanted for less than 5 years [13]. Since the inception of our program, 101 annealed HXLPE liners have been retrieved in our orthopaedic implant retrieval program. Sixty-three highly crosslinked and annealed liners (Crossfire; Stryker Orthopaedics; implanted 2.2 ± 1.4 years; range, 0–5 years) met the inclusion criterion for the study (namely, in vivo for less than 5 years). Crossfire is fabricated from GUR 1050 polyethylene rod stock that has been gamma-irradiated with 75 kGy and subsequently annealed at 130° C. After fabricating the liners from the irradiated and annealed bar stock, Crossfire liners are barrier-packaged and gamma radiation-sterilized in nitrogen (30 kGy) for a total nominal radiation dose of 105 kGy [17]. The annealed cohort consisted of liners from two designs (Omnifit [n = 28] and Trident [n = 35]; Stryker Orthopedics), which differed primarily in terms of their locking mechanisms [13]. We excluded one liner from the annealed cohort that was fabricated from an early design (System 12). In a previous study [13], we found no difference between Omnifit and Trident Crossfire liners in terms of their reasons for revision, in vivo oxidation behavior, or mechanical properties after up to 6 years in vivo. Consequently, we incorporated both of these types of retrieved liners into a single group for the annealed cohort. Sterilization information was traceable in 57 of 63 (90%) retrieved liners. The mean shelf life was 0.6 ± 0.8 years (range, 0.1–6.4 years).

The annealed liners had smaller inner diameters than the sequentially annealed group. Retrieved liners from the annealed cohort had inner diameters of 28 mm (n = 27), 32 mm (n = 29), or 36 mm (n = 7; median inner diameter = 32 mm; p < 0.001). The head material was a cobalt-chrome alloy in 73% (46 of 63) of the cases, a ceramic in 17% (11 of 63) of the cases (zirconia in seven, alumina in four), and the head material was unable to be ascertained in six cases. The measured thickness of the liners, in unworn locations, ranged from 5 to 15 mm. The measured thickness was greater in the annealed group when compared with the sequentially annealed group (mean difference, 2 mm; p < 0.001). The outer diameter of the acetabular shells varied between 46 and 70 mm (median, 54 mm).

Clinical data, including patient activity level, were collected from the medical records for both cohorts (Table 1). Patient activity level was assessed in 70% (86 of 122) of the study cohort and 71% (45 of 63) of the control cohort using the UCLA activity scale ranging from 1 to 10. Patients were asked in a questionnaire to assess their activity level before the onset of symptoms leading to revision surgery. The patient characteristics in the two cohorts did not differ in terms of sex (p = 0.91) and body mass index (p = 0.15) (Table 1). The retrieved control cohort was slightly older (mean difference, 5 years; p = 0.01) and slightly more active (median difference, 1; p = 0.048; Table 1). Forty percent (44 of 110) of the revised components from the sequentially annealed cohort and 52% (28 of 54) from the annealed cohort were used in patients who had a history of at least one previous revision surgery. Reasons for revision were assessed based on medical records, radiographs, and examination of the retrieved components.

**Table 1:** Summary of patient demographics for the study and control cohorts

Cohort	Number	Age (years)*	Sex (percent female)	Body mass index (kg/m^2^)*	Implantation time (years) (range)	Maximum UCLA score (range)
Sequentially annealed	122	58 ± 14	59	30 ± 8	1.2 ± 1.2 (0.0–5.0)	4 (1–8)
Annealed (control)	63	63 ± 12	59	28 ± 6	2.2 ± 1.4 (0.0–4.9)	5 (2–10)

* Mean ± SD.

### Analysis of Oxidation

Thin sections (approximately 200 µm) were taken from the superoinferior axis of the liners using a sledge microtome (Leitz 1400, Wetzlar, Germany). Absorbed lipids have been shown to interfere with the oxidation analysis [12]; thus, lipids were extracted from the HXLPE slices before analysis by boiling in heptane for 6 hours. Using transmission Fourier transform infrared spectroscopy, 3-mm line profiles were taken perpendicular to the surface of each region of interest at 100-μm increments. Regions of interest included the bearing surface, the backside surface, the rim, and the locking mechanism of both the superior and inferior portions of the liner. An oxidation index was calculated in accordance with ASTM 2102 [1].

### Analysis of Linear Penetration, Mechanical Behavior, and Rim Damage

As an indicator of wear, linear femoral head penetration was assessed directly using a calibrated digital micrometer (accuracy, 0.001 mm). The thickness of the liners was measured in the loaded and unloaded regions. We excluded liners implanted less than 1 year from the penetration analysis because creep may dominate femoral head penetration during the first year after implantation [20]. For the 55 study and 45 control liners that were implanted for longer than 1 year, we calculated an average femoral head penetration rate (mm/year) by dividing the measured head penetration by the implantation time, as described previously [10]. For mechanical properties, we relied on the small punch test (ASTM F2183) [2] using the same sampling protocol as our previous retrieval studies [16]. Briefly, in both superior and inferior regions of the retrieved liners, small punch specimens were sampled near the surface (0–0.5 mm) and below the surface (1.5–2 mm). Thus, for each liner, at least four specimens were tested (depending on material availability), which led to a total of 889 small punch tests performed on the 185 retrievals. From the force-displacement curve, four metrics were calculated: peak load, ultimate load, ultimate displacement, and energy to failure. Ultimate load is a measure of the ultimate strength of the polymer and was chosen as the primary metric in this study.

Liners were inspected using optical microscopy for evidence of rim damage, subsurface fatigue, and cracking, consistent with previous studies [5, 13]. Damage mechanisms along the rim were visually scored using a semiquantitative scale similar to the Hood method [11]. The rim was inspected and scored as a single entity. Specifically, we inspected for the presence of abrasion, burnishing, delamination, embedded debris, plastic deformation, pitting, and scratching. A score of 0 was given if the damage mechanism was not present or if the damage appeared to be caused during removal (eg, scratching from an osteotome). A score of 1 was given if the damage covered 1% to 10% of the rim surface. A score of 2 was given for damage that covered 10% to 50% of the rim surface. Damage that covered more than 50% of the rim surface was given a score of 3. Starting in 2003, we also began routinely analyzing retrieved liners using micro-CT. We reviewed 84 of 122 of the sequentially annealed liners and 38 of 63 of the annealed liners using micro-CT reconstructions for evidence of rim damage or impingement. When we observed rim damage, either in optical micrographs or micro-CT data sets, we sought to establish a root cause based on prerevision radiographs and medical records.

### Statistical Analysis Methodology

Distributions of continuous variables were tested for normality using the Shapiro-Wilk test and were generally found to be nonnormal. Thus, differences between the sequentially annealed and annealed cohorts were evaluated using the Wilcoxon test. Differences among categorical variables (gender and presence of rim damage) were assessed using contingency table analysis. For correlation statistics, we relied on the Spearman’s rank correlation test. All statistics were performed using commercial statistical software (JMP 10.0; SAS Institute, Cary, NC, USA).

## Results

**Fig. 1:**
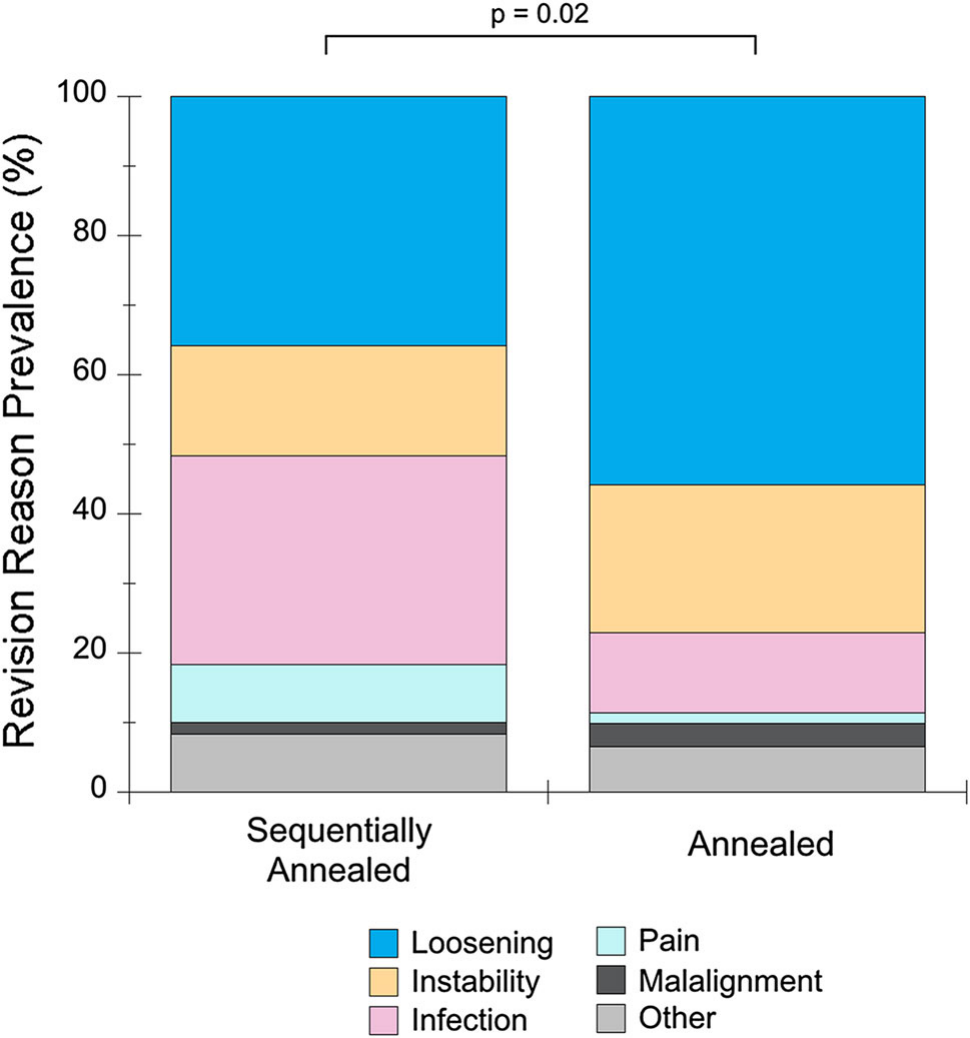
The revision reasons for sequentially annealed and annealed HXLPE liner cohorts were different. Loosening and instability were more prevalent in the annealed HXLPE liner cohort, whereas infection was more prevalent in the sequentially annealed HXLPE liner cohort.

The study cohort of sequentially annealed liners was primarily revised for loosening (43 of 122 [35%]), infection (36 of 122 [30%]), and instability (19 of 122 [16%]). This was different (p = 0.02) from the control cohort of annealed liners that was revised for loosening (55%), instability (21%), and infection (11%) (Fig. 1). None of the liners in either cohort was revised for polyethylene wear or mechanical failure of the polyethylene.

**Fig. 2:**
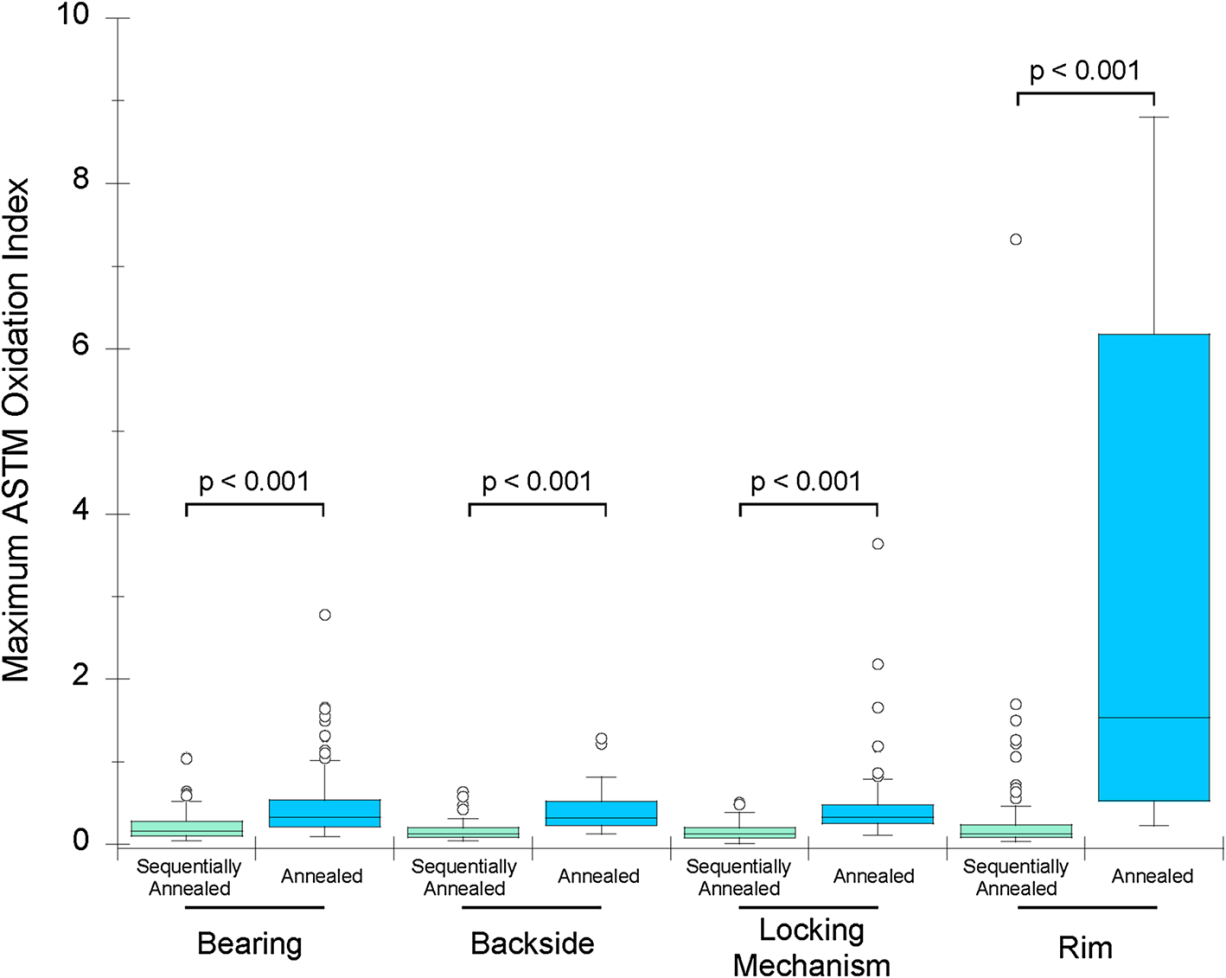
Sequentially annealed HXLPE liners exhibited lower maximum oxidation indices at all measured locations when compared with the annealed HXLPE liners (p < 0.001).

Oxidation indices (OIs) of the sequentially annealed liners were lower than annealed liners at the bearing surface (mean OI difference = 0.3; p < 0.001; Fig. 2), the backside surface (mean OI difference = 0.2; p < 0.001), the locking mechanism (mean OI difference = 0.3; p < 0.001), and the rim (mean OI difference = 2.6; p < 0.001; Fig. 2). Regional variation was observed in both cohorts, particularly at the rim of the liners, which had the highest oxidation. In the annealed cohort, the rim had higher oxidation indices than the bearing surface (mean OI difference = 2.4; p < 0.001), the backside surface (mean OI difference = 2.6; p < 0.001), and the locking mechanism (mean OI difference = 2.5, p < 0.001). The regional differences in the sequentially annealed liners were less pronounced. The rim had higher oxidation indices than the bearing surface (mean OI difference = 0.2; p = 0.01) and the backside surface (mean OI difference = 0.2; p = 0.03). Additionally, the bearing surface had higher oxidation indices than the backside surface (mean OI difference = 0.1; p < 0.001) and the locking mechanism (mean OI difference = 0.1; p < 0.001). Oxidation was not correlated with liner thickness in either HXLPE cohort (p > 0.14). Oxidation was positively correlated with implantation time at the rim of the annealed liners (Rho = 0.64; p < 0.001). For the sequentially annealed cohort, implantation time was positively correlated with oxidation indices at all measured locations (Rho = 0.21–0.43; p ≤ 0.02). Shelf life was not correlated with oxidation in the annealed cohort at any measured location (p ≥ 0.40). For the sequentially annealed liners, shelf life was positively correlated with oxidation indices at the backside (Rho = 0.23; p = 0.02), the locking mechanism (Rho = 0.25; p = 0.01), and the rim (Rho = 0.25; p < 0.01), but not the articulating surface (Rho = 0.13; p = 0.19).

**Fig. 3A–B:**
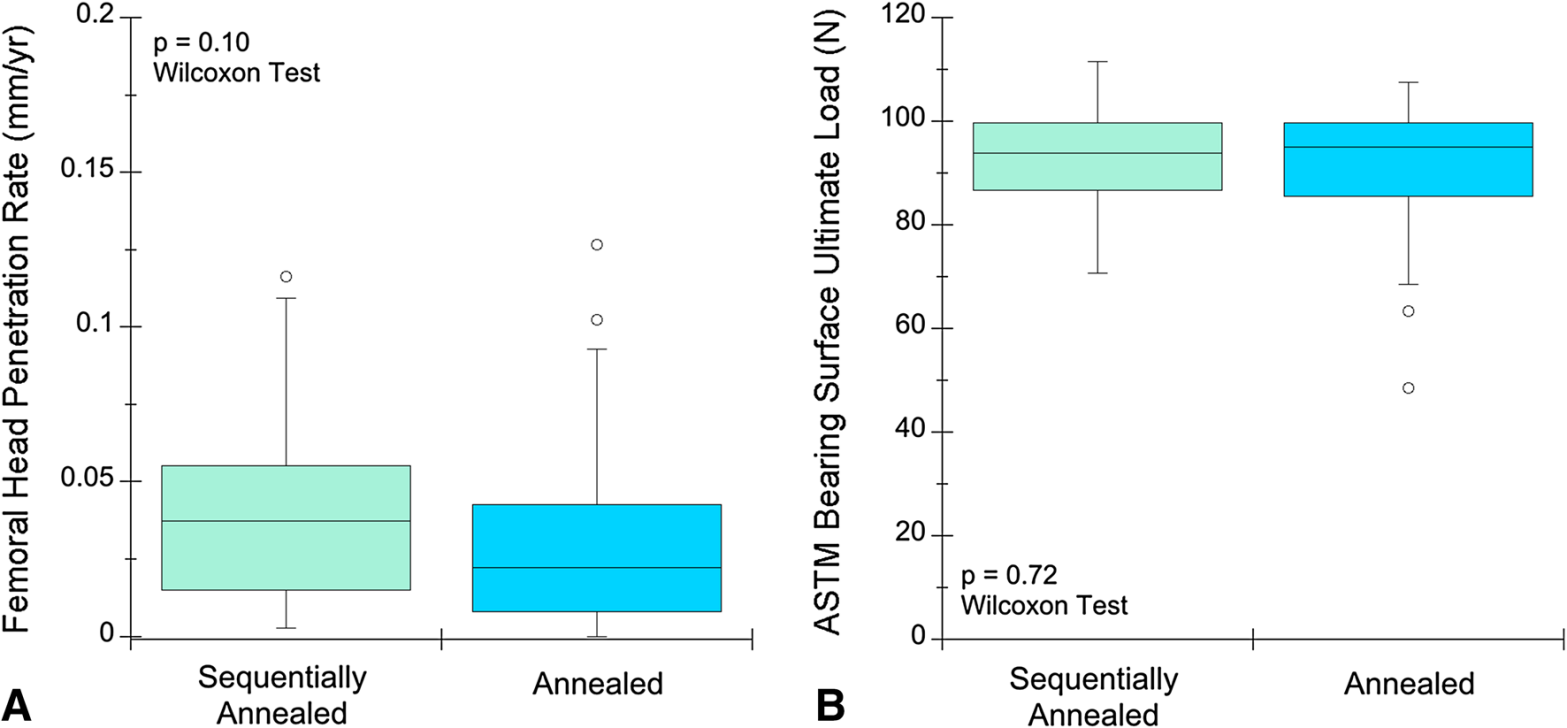
The femoral head penetration rates (**A**; p = 0.10) and small punch ultimate strength of the bearing surface (**B**; p = 0.72) were not different between the sequentially annealed HXLPE and annealed HXLPE liner cohorts.

Femoral head penetration rate and mechanical behavior of the sequentially annealed and annealed HXLPE liners were similar. No differences were detected in linear penetration rates between the annealed and sequentially annealed liners (p = 0.10; Fig. 3A). The median penetration rate was 0.04 mm/year (range, 0.0–0.12 mm/year; interquartile range [IQR], 0.04 mm/year) for the sequentially liners and 0.02 mm/year (range, 0.0–0.13 mm/year; IQR, 0.04 mm/year) for the annealed liners. Ultimate strength at the bearing surface of the HLXPE was not different between sequentially annealed and annealed cohorts (p = 0.72; Fig. 3B). The median ultimate load evaluated near the superior bearing surface was 94 N (IQR, 13 N) for the sequentially annealed liners and 95 N (IQR, 14 N) for the annealed liners.

**Fig. 4A–B:**
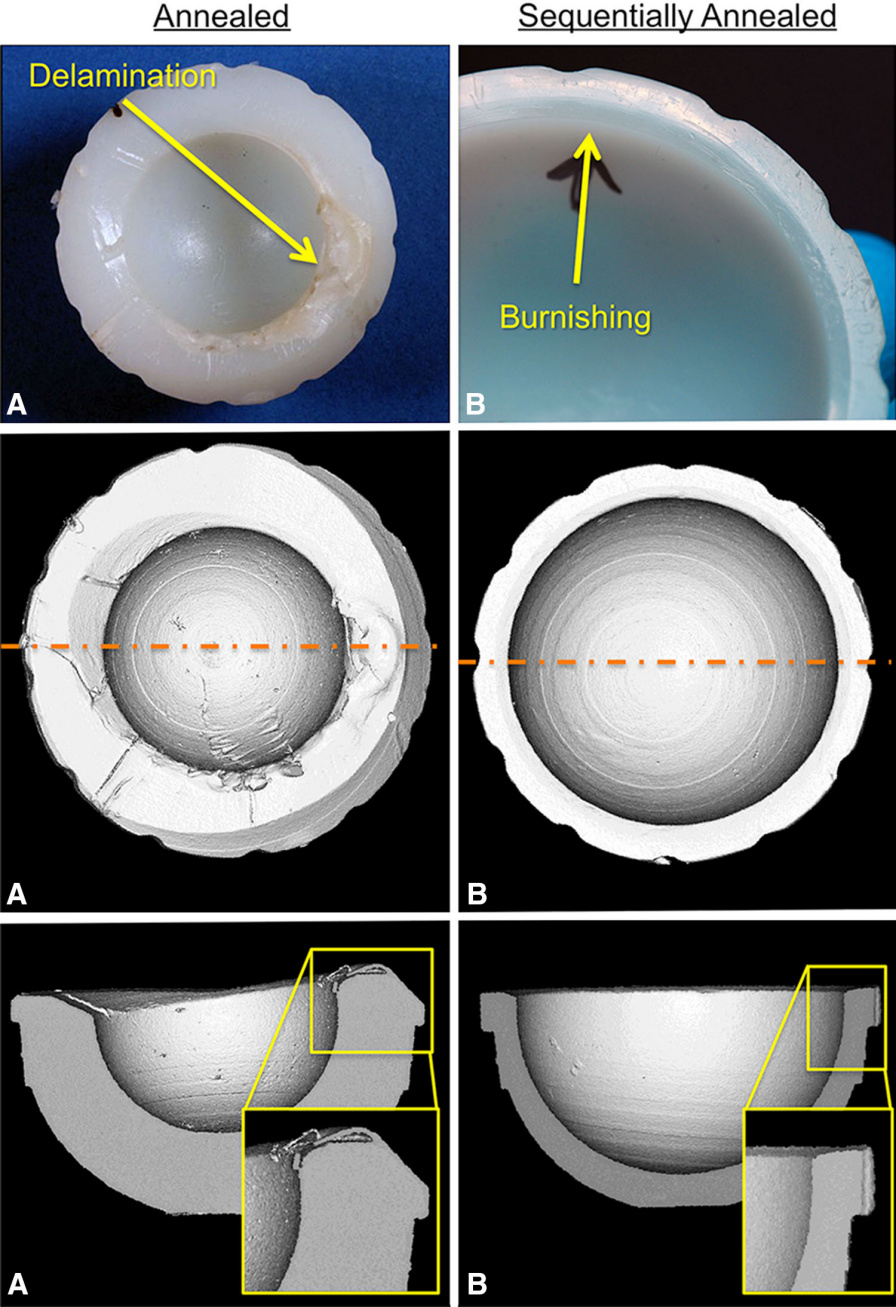
Examples of rim damage in an annealed (**A**) and a sequentially annealed (**B**) HXLPE liner are shown. Note the delamination of the rim of the annealed HXLPE liner.

Rim damage (ie, burnishing, delamination, etc) was observed on 12 of 122 (10%) of sequentially annealed and six of 63 (10%) of the annealed liners. With the numbers available, the prevalence of rim damage was not different between the two cohorts (p = 0.95); however, the type of damage mechanism varied between the cohorts (Table 2). For the sequentially annealed cohort that presented with rim damage, the damage was primarily in the form of burnishing (median score of 2) and scratching (median score of 1), which appeared to be a result of femoral neck impingement or from articulation with the femoral head during dislocation (Fig. 4). There were no instances of delamination in the sequentially annealed cohort. Inspection of the three-dimensional micro-CT data sets revealed no instances of internal cracking at the rim of sequentially annealed liners (Fig. 4). The annealed cohort had similar damage scores for burnishing and scratching; however, this cohort also had several cases of delamination of the rim (Fig. 4; median delamination score = 1.5). In one case with delamination, subsurface cracking could be observed through the micro-CT data sets that covered an arc of approximately 180°. The remaining delamination cases were destructively tested before routine micro-CT scanning of polymer implants at our institution.

**Table 2:** Damage mechanism scores for implants that had evidence of damage to the rim*

Implant ID	Liner material	Delamination score	Burnishing score	Scratching score	Abrasion score
0086	Annealed	0	1	1	0
0343	Annealed	1	2	0	1
0345	Annealed	3	1	3	0
0578	Annealed	2	1	1	0
0193	Annealed	2	1	2	0
0018	Annealed	0	0	0	2
0676	Sequentially annealed	0	2	1	0
0838	Sequentially annealed	0	1	1	0
0751	Sequentially annealed	0	2	1	0
0753	Sequentially annealed	0	2	1	0
0903	Sequentially annealed	0	2	1	0
0914	Sequentially annealed	0	1	0	1
0926	Sequentially annealed	0	1	2	0
1068	Sequentially annealed	0	2	1	0
1080	Sequentially annealed	0	2	2	0
0218	Sequentially annealed	0	1	2	0
0327	Sequentially annealed	0	1	1	0
0364	Sequentially annealed	0	2	2	0
Annealed summary [mean (median)]		1.3 (1.5)	1.0 (1.0)	1.2 (1.0)	0.5 (0.0)
Sequentially annealed summary [mean (median)]		0.0 (0.0)	1.6 (2.0)	1.3 (1.0)	0.1 (0.0)

* The rim of each liner was scored as a single entity. The maximum score for each damage mechanism is 3. Note: embedded debris, plastic deformation, and pitting were not observed on any rim surface.

## Discussion

HXLPEs were clinically introduced for THAs to improve wear resistance of the polyethylene liners and, thus, to reduce the incidence of polyethylene wear debris-induced osteolysis. Sequentially annealed HXLPE was introduced in 2005 to improve the oxidative resistance of single-dose annealed HXLPE. Although in vitro testing has demonstrated reduced oxidation, there is little known about the in vivo performance of sequentially annealed HXLPE used in THA. In this study, we compared the reasons for revision, the polyethylene properties, and the rim damage among first- and second-generation annealed HXLPEs acetabular liners used in THA. The HXLPEs in this study had similar femoral head penetration rates and mechanical properties (as assessed through small punch testing). However, sequentially annealed HXLPE had lower oxidation indices than annealed HXLPE in all measured locations.

There were several limitations to this study. The sequentially annealed HXLPE liners were only implanted for 1 to 2 years on average and at most 5 years; it will remain necessary to continue the analysis long term to assess the oxidative resistance, mechanical properties, and wear performance of this material. Although long-term followup would admittedly be useful, recent research [3] indicates that patient activities are greatest during the first 5 years after implantation, and thus, the study period likely captures the time period during which patients are expected to be most active. Nevertheless, the results from this study should not be extrapolated to the long-term performance of sequentially annealed HXLPE. Therefore, more research is necessary to assess the intermediate and long-term tribological and oxidative behavior of sequentially annealed HXLPE. Also, the retrieved liners were collected at revision surgery and do not necessarily capture the behavior of well-functioning implants. On the other hand, there is no reason to suspect that revision would alter the natural progression of in vivo oxidation or mechanical degradation of the HXLPE materials, and we mitigated against any ex vivo aging after removal from the body by cryogenically storing the explants after removal. Finally, in addition to the number of annealing steps, there are other differences between the materials including: sterilization dose, sterilization method, and UHMWPE resin. Therefore, this is not a direct comparison between single- and three-step annealing and the data should not be interpreted as such.

In this study, the predominant reasons for revision were loosening, infection, and instability, albeit in different proportions within the cohorts. Specifically, infection occurred in a larger percentage of patients in the sequentially annealed cohort. This may be attributable in part to the timing of the release of new materials and the inception of our retrieval program. When our program began, annealed HXLPE liners were already clinically available for several years. By the time sequentially annealed HXLPE liners were clinically available, our program was routinely receiving retrievals from several clinical centers. Therefore, short-term revision reasons (ie, infection) may be more represented in the sequentially annealed cohort. None of the liners in either cohort was revised for wear or mechanical failure of the polyethylene. These findings are similar to both a recent retrieval study on the revision reasons of first-generation HXLPEs [18] and a nationally representative administrative database study of revision THA [4]. In both of these studies, the three predominant reasons for revision were mechanical loosening, instability, and infection.

The results of our study support that second-generation sequentially annealed HXLPE has improved oxidative properties (as seen by a reduction in maximum oxidation index) compared with first-generation annealed HXLPE. This improvement was observed at all measured regions of the liners. The improvement of oxidative behavior is similar to what was seen in a previous study, which reported mean ASTM oxidation indices of 0.31 and 0.11 [5] at the bearing and unloaded edges, respectively [6]. However, we observed a different regional pattern in the sequentially annealed HXLPE liners in this study. The bearing, backside, and locking mechanism regions of the sequentially annealed HXLPE liners had low oxidation indices (mean OI approximately 0.1–0.2), whereas the rim of the liners had slightly higher indices (mean OI = 0.3). For the annealed cohort, the regional variations are similar in pattern but were more pronounced with mean differences in oxidation indices of approximately 2.5. The difference in oxidation patterns is likely the result of the conforming nature of the liners, which reduces contact stresses and may limit the access of oxygen-containing fluids to the bearing surface, whereas the rim is exposed to oxygen-containing fluids and tissues. Oxidation indices of sequentially annealed liners were positively correlated with implantation time and shelf life. However, the levels of oxidation were generally low and below the levels necessary typically thought to begin degrading the mechanical and tribological properties of UHMWPE (ASTM oxidation index > 1) and were not associated with the clinical performance of the sequentially annealed HXLPE cohort in this study.

HXLPE materials were developed specifically to reduce polyethylene wear and the subsequent wear debris-induced osteolysis. In the current study, the femoral head penetration rates of both cohorts were well below the proposed osteolysis threshold (approximately 0.1 mm/year) [9]. However, the components in this study were collected at revision surgery; therefore, these data may not reflect femoral head penetration rates in well-functioning implants with proper positioning. Radiographic studies of femoral head penetration have consistently shown that annealed HXLPE has a reduced femoral head penetration rate compared with conventional (gamma radiation sterilized in inert gas or vacuum packaging) UHMWPE [7, 20, 21]. The mean penetration rates for conventional UHMWPE in these radiographic studies ranged from 0.13 to 0.20 mm/year. Similarly, the femoral head penetration rates in the current study were lower than previously reported for conventional UHMWPEs. With the available numbers, no difference was detected in penetration rates between the annealed and sequentially annealed cohorts. Given the magnitudes observed here (on the order of 30 μm/year), a post hoc power analysis revealed that we only had 18% power to identify significant differences. Nearly 1000 retrieved liners would be required to determine whether such a difference was significant. This suggests that the differences between these two implant designs in terms of wear—if any—are likely to be small and perhaps clinically unimportant. With respect to mechanical properties, prior studies have found that the ultimate load (as assessed by the small punch test) is higher in HXLPEs than in conventional UHMWPE [19]. The results in this study are similar to values seen in previous retrieval studies of annealed HXLPE [13, 16, 19]. Previously, the ultimate load of UHMWPE has been found to decrease with implantation and to be associated with an increase in oxidation [15]. Collectively, the penetration rate and ultimate load results from this study suggest that any oxidation that was observed was not sufficient to degrade these properties to any appreciable degree in either annealed of sequentially annealed HXLPE, at least when implanted for short-term durations.

Recently, there have been reports of fatigue damage observed at the rims of first-generation annealed HXLPE [5, 13]. Currier et al. [5, 13] reported on 12 retrieved annealed HXLPE liners (mean implantation time, 2 years; range, 0.1–5 years) from five different institutions. They observed delamination in three of 12 (25%) cases and unintended articulation with the rim (through dislocation or impingement) in five of 12 (42%) liners. In this study, both the annealed and sequentially annealed cohorts had a lower prevalence of rim damage (approximately 10% in both cohorts) than previously reported [5]. Additionally, the sequentially annealed cohort appeared to be more resistant to fatigue damage modes, namely delamination. It has been observed that delamination in first-generation annealed HXLPE liners was correlated with the combination of oxidation and unintended rim articulation (either in the form of dislocation or neck impingement) [5]. Therefore, the improved resistance to delamination observed in sequentially annealed HXLPE liners might be the result of the better oxidative resistance demonstrated in this study.

Over the past decade, several modifications have been introduced in HXLPE to improve the oxidative and/or fatigue properties of the first generation of these materials. These include thermal treatments (ie, sequential annealing) as well as the inclusion of antioxidant additives. As new materials are introduced into clinical practice, it is important to monitor the in vivo performance of HXLPE materials to ensure no unintended outcomes occur that were not foreseen in in vitro testing. In this study, we compared the revision reasons, oxidative behavior, and small punch mechanical behavior of second-generation sequentially annealed HXLPE liners with those of first-generation annealed HXLPE liners in THA. The sequentially annealed HXLPE had similar mechanical properties (as assessed through femoral head penetration measurements and the small punch test) of annealed HXLPE. Additionally, the sequentially annealed HXLPE liners also appeared to be more resistant to oxidative degradation compared with the annealed HXLPE liners. The oxidation indices of the sequentially annealed liners did have a positive correlation with implantation time and shelf life; however, the levels of oxidation were not high enough to negatively impact the measured mechanical properties. Future retrieval studies, with long-term implanted liners, will be useful for documenting the natural history of oxidation in sequentially annealed HXLPE.

## References

[1] ASTM Standard F2102-13. *Standard Guide for Evaluating the Extent of Oxidation in Polyethylene Fabricated Forms Intended for Surgical Implants.* West Conshohocken, PA, USA: ASTM International; 2013.

[2] ASTM Standard F2183-02(2008). *Standard Test Method for Small Punch Testing of Ultra-high Molecular Weight Polyethylene Used in Surgical Implants.* West Conshohocken, PA, USA: ASTM International; 2008.

[3] Battenberg AK, Hopkins JS, Kupiec AD, Schmalzried TP (2013). The 2012 Frank Stinchfield Award: Decreasing patient activity with aging: implications for crosslinked polyethylene wear. Clin Orthop Relat Res..

[4] Bozic KJ, Kurtz SM, Lau E, Ong K, Vail TP, Berry DJ (2009). The epidemiology of revision total hip arthroplasty in the United States. J Bone Joint Surg Am..

[5] Currier BH, Currier JH, Mayor MB, Lyford KA, Collier JP, Van Citters DW (2007). Evaluation of oxidation and fatigue damage of retrieved crossfire polyethylene acetabular cups. J Bone Joint Surg Am..

[6] Currier BH, Van Citters DW, Currier JH, Carlson EM, Tibbo ME, Collier JP (2013). In vivo oxidation in retrieved highly crosslinked tibial inserts. J Biomed Mater Res B Appl Biomater..

[7] D’Antonio JA, Manley MT, Capello WN, Bierbaum BE, Ramakrishnan R, Naughton M, Sutton K (2005). Five-year experience with Crossfire highly cross-linked polyethylene. Clin Orthop Relat Res..

[8] Dumbleton JH, D’Antonio JA, Manley MT, Capello WN, Wang A (2006). The basis for a second-generation highly cross-linked UHMWPE. Clin Orthop Relat Res..

[9] Dumbleton JH, Manley MT, Edidin AA (2002). A literature review of the association between wear rate and osteolysis in total hip arthroplasty. J Arthroplasty..

[10] Gomez-Barrena E, Li S, Furman BS, Masri BA, Wright TM, Salvati EA (1998). Role of polyethylene oxidation and consolidation defects in cup performance. Clin Orthop Relat Res..

[11] Hood RW, Wright TM, Burstein AH (1983). Retrieval analysis of total knee prostheses: a method and its application to 48 total condylar prostheses. J Biomed Mater Res..

[12] James SP, Blazka S, Merrill EW, Jasty M, Lee KR, Bragdon CR, Harris WH (1993). Challenge to the concept that uhmwpe acetabular components oxidize in-vivo. Biomaterials..

[13] Kurtz SM, Austin MS, Azzam K, Sharkey PF, MacDonald DW, Medel FJ, Hozack WJ. Mechanical properties, oxidation, and clinical performance of retrieved highly cross-linked Crossfire liners after intermediate-term implantation. *J Arthroplasty.* 2010;25:614–623.e611–612. 10.1016/j.arth.2009.04.022 PMC2876196 19520545

[14] Kurtz SM, Gawel HA, Patel JD (2011). History and systematic review of wear and osteolysis outcomes for first-generation highly crosslinked polyethylene. Clin Orthop Relat Res..

[15] Kurtz SM, Hozack W, Marcolongo M, Turner J, Rimnac C, Edidin A (2003). Degradation of mechanical properties of UHMWPE acetabular liners following long-term implantation. J Arthroplasty..

[16] Kurtz SM, Hozack W, Turner J, Purtill J, MacDonald D, Sharkey P, Parvizi J, Manley M, Rothman R (2005). Mechanical properties of retrieved highly cross-linked crossfire liners after short-term implantation. J Arthroplasty..

[17] Kurtz SM, Manley M, Wang A, Taylor S, Dumbleton J (2002). Comparison of the properties of annealed crosslinked (Crossfire) and conventional polyethylene as hip bearing materials. Bulletin..

[18] Kurtz SM, Medel FJ, MacDonald DW, Parvizi J, Kraay MJ, Rimnac CM (2010). Reasons for revision of first-generation highly cross-linked polyethylenes. J Arthroplasty..

[19] Macdonald D, Sakona A, Ianuzzi A, Rimnac CM, Kurtz SM (2011). Do first-generation highly crosslinked polyethylenes oxidize in vivo?. Clin Orthop Relat Res..

[20] Martell JM, Verner JJ, Incavo SJ (2003). Clinical performance of a highly cross-linked polyethylene at two years in total hip arthroplasty: a randomized prospective trial. J Arthroplasty..

[21] Rajadhyaksha AD, Brotea C, Cheung Y, Kuhn C, Ramakrishnan R, Zelicof SB (2009). Five-year comparative study of highly cross-linked (crossfire) and traditional polyethylene. J Arthroplasty..

[22] Wang A, Zeng H, Yau SS, Essner A, Manely M, Dumbleton J (2006). Wear, oxidation and mechanical properties of a sequentially irradiated and annealed UHMWPE in total joint replacement. J Phys D Appl Phys..

[23] Wannomae KK, Bhattacharyya S, Freiberg A, Estok D, Harris WH, Muratoglu O (2006). In vivo oxidation of retrieved cross-linked ultra-high-molecular-weight polyethylene acetabular components with residual free radicals. J Arthroplasty..

